# Care Farming Program for Family Health: A Pilot Study with Mothers and Children

**DOI:** 10.3390/ijerph17010027

**Published:** 2019-12-18

**Authors:** A-Young Lee, Seon Ok Kim, Gyung Mee Gim, Dae Sik Kim, Sin-Ae Park

**Affiliations:** 1 Department of Environmental Health Science, Sanghuh College of Life Science, Konkuk University, Seoul 05029, Korea; danapre0302@gmail.com; 2 Department of Bio and Healing Convergence, Graduate School, Konkuk University, Seoul 05029, Korea; kso0804@naver.com; 3 Research Policy Bureau, Rural Development Administration, Jeonju 54875, Korea; gimgm@rda.go.kr; 4 Department of Agricultural and Rural Engineering, Chungnam National University, Daejeon 34134, Korea; drkds19@cnu.ac.kr

**Keywords:** green care, gardening, horticultural therapy, social farming, socio horticulture

## Abstract

We designed a pilot study to develop a family interaction model-integrated a care farming program with mother-child pairs as the participants. In this pilot study, we aimed to assess the effects of the care farming program on communication skills and psychological health in families. Sixteen mother-child pairs in Sejong, South Korea participated in this study. The families participated in a care farming program once a week for six weeks (90 min per session) between May and July 2018. The care farming program was developed based on parenting education skills, strengths-based cognitive behavioral therapy, and the emotional intelligence model; the result was a family interaction model intended to improve communication and psychological health among mothers and children. The program consisted of gardening activities such as making a garden plot, planting transplants, harvesting, and cooking the harvested crops. Upon completion of the six-session program, we evaluated communication with the Parent-Children Communication Inventory, depression with the Beck Depression Inventory, and resilience with the Connor–Davidson Resilience Scale among the mothers. We also evaluated emotional intelligence among the children with the Emotional Intelligence Scale. According to post-intervention results, mothers showed significantly increased resilience, improved communication skills with their child, and decreased depression, while children showed significantly improved emotional intelligence (*p* < 0.05). Despite the study’s limitation in establishing causality between the care farming program and the observed effects on family health, the care farming program clearly contributed to the observed improvements of mother-child communication skills, mothers’ psychological health, and children’s emotional intelligence, which in turn improved overall family health.

## 1. Introduction

In contemporary society, the nature and composition of families are constantly evolving due to factors such as lower marriage and fertility rates, increased opportunities for women’s social activities, population aging, and the rapid development of social economy. In most member countries of the Organization for Economic Co-operation and Development, the average household size has dropped from 2.8 people in the mid-1980s to 2.6 people in the mid-2000s [1,2]. In South Korea in 2015, the average household size was 2.8 people and 94.3% of families were considered to be a type of “small family” (not including extended relatives); this includes nuclear families, couples, single-parent families, grandparent-headed families, and single-person households [3]. Smaller and more nuclear families have become commonplace, thereby weakening family functions such as support and care for family members and consequently increasing the frequency of family conflict [4]. Interventions for preventing and resolving family conflicts are therefore urgently needed [5].

Parent-child interactions are an important factor in maintaining family functioning and promoting family health. Families today often spend less time together than in the past, which can result in poorer communication and weaker familial bonds [6]. In South Korea, interactions between family members are becoming less frequent as families are becoming smaller in size and work-centric culture is spreading more widely in modern society. The average amount of time spent together as a family was 171 min per day in 2009 and 127 min per day in 2014, demonstrating a 25.7% decrease in the amount of time for family interactions [7]. Positive parent-child interactions contribute to improving coping competence and behavioral adjustment in children and increasing satisfaction and self-efficacy in parents [8,9,10]. Behavior-oriented parent training can improve family interactions through the practice of positive parenting, thus effectively preventing and resolving psychological and behavioral problems within families such as parenting stress, child abuse, and misbehavior of children [11,12,13]. Research has shown that cooperation between parents and children positively affects children’s psychological characteristics such as well-being, achievement, and satisfaction with their parents [14].

“Care farming” is defined in many ways and there is still debate about the most appropriate definition. In South Korea, the Rural Development Administration defines care farming as a healing service with activities and products that utilize agricultural and rural resources to provide various health benefits [15]. In many European countries, the benefits of care farming have been recognized, and both formal and informal care farming systems have been organized, developed, and tailored to cultural conditions and needs [16,17,18]. In South Korea, the field of agriculture has expanded more broadly from simply production-oriented agriculture, and interest in care farming has increased due to the government’s recent “Sixth Industrialization of Agriculture” policy [15]. In order to create and develop a suitable care farming system in South Korea during this preliminary stage, studies have been conducted to investigate the awareness of and need for care farming, demand and operation status of care farms, development of care farming activities for various clients, and viable ways to establish and promote the concept of care farming [19,20]. These activities take place in natural settings offered by care farms. As such, care farms are the core element of care farming, not only functioning as agriculturally-productive farms, but also providing space to care, cure, or promote the health of various clients [21]. Care farming offers improvement of participants’ well-being, revitalization of rural landscapes, and conservation of agroecosystems [22,23]. Specifically, care farming improves participants’ well-being via its beneficial effects on a wide range of components such as physical, psychological, cognitive, social, educational, and behavioral aspects [24]. Care farming programs in which families participate together have the potential to improve factors related to family functioning, such as family adaption and resilience [25]. Studies have shown that by participating in care farming, parents experience beneficial effects associated with improved family interactions and psychological health [26,27,28]. For example, a 24-week care farming program was shown to improve mother-child communication and help mitigate stress among mothers [26]. Another study reported improved parent self-efficacy and self-esteem and mitigated parenting stress among mothers who participated in a six-week care farming program, as compared to a control group [28]. In other studies, children were found to have improved overall health outcomes, including physical and physiological (e.g., physical activity and cortisol levels), psychological (e.g., emotional intelligence, eco-friendly attitude, and self-esteem), cognitive (e.g., attention and concentration, and creativity), social (e.g., peer relationships and social ability), educational (e.g., scientific attitude and exploration ability), and behavioral (e.g., dietary habits) improvements [24,29,30,31,32,33,34,35,36,37,38]. However, most studies conducted to date have only explored the effects of care farming programs for individuals such as adult women or children, and have not focused on the relationships or interactions between family members within a family care farming program.

Typically, parental trainings meant to improve family interactions are limited to providing lessons only to parents rather than providing practical, hands-on training for both parents and their children [13]. In contrast to this standard training method, the care farming program developed and analyzed in this study actively involved all parent and child participants in care farming activities, monitored their interactions, and assessed changes in their communication. They were given objective-oriented activities to increase interactions with each other under certain program circumstances. Thus, we feel an interactive care farming program is likely to be more effective than standard parent training in improving psychological health indicators such as depressive symptoms and resilience in mothers and emotional intelligence in children, which would contribute to improving overall family health. We therefore hypothesized that a care farming program that promotes and integrates family interaction into its activities would provide positive effects on family communication and psychological health associated with improvements in overall family health. To test this hypothesis, we developed a six-session care farming program in which mothers and their children participated together. We then examined the effects of the program on family interactions between the parents and children and investigated respondents’ degrees of satisfaction with participation in care farming activities. This study aimed to evaluate the suitability of such a program as a parental training intervention to improve family health in South Korea.

## 2. Materials and Methods

### 2.1. Participants and Experimental Design

To recruit participants, flyers including information about the study’s objective, a description of the program, a timeline of the schedule, an outline of requirements, and a registration form were distributed at the S Healthy Family Support Center in Sejong, South Korea. The inclusion criteria for participants were that they were mothers with a child under the age of seven. Sixteen families (16 mothers and 16 children) volunteered and subsequently provided consent to participate in this study. The participating mothers answered questionnaires to provide demographic data such as gender, age, and highest education level, as well as information on what leisure activities they participated in and whether they had prior experience in a care farming program.

The average ages of mothers and children were 37.81 years old (SD = 3.45) and 3.31 years old (SD = 0.95), respectively (Table 1). Only one family had previous experience of participating in a care farming program. This study featured a one group pretest–post-test design. This study was approved by the institutional review board (7001355-201803-HR-251).

### 2.2. Developing a Care Farming Program for Family Health

The care farming program lasted six sessions and consisted of gardening activities such as making a garden plot, sowing seeds, planting transplants, harvesting, and cooking the harvested crops. Seasonal crops were grown (e.g., lettuce, chicory, and spinach).

**Figure 1:**
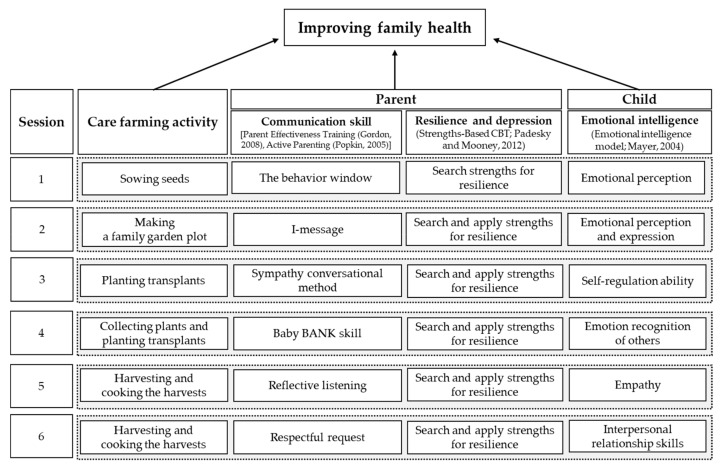
Development of the care farming program for improving family health.

Each session of the care farming program was designed to strengthen communication skills between mothers and children, improve resilience and reduce depressive symptoms in mothers, and increase emotional intelligence in children (Figure 1). In each session, mothers gave a lesson on communication skills to their children and had the opportunity to practice the lesson with their children during the gardening activities. To improve mothers’ communication skills, parent effectiveness training was implemented to promote positive interactions [39,40]. These lessons included the following: (1) the behavior window, to contemplate acceptance/unacceptance of their children’s behaviors and practice appropriate communication skills with their children; (2) I-messages, to allow parents to convey their emotions to their children; (3) the sympathy conversational method, to better sympathize with their children’s emotions; (4) the Baby BANK skill, to acknowledge their children’s achievements and encourage certain positive behaviors; (5) reflective listening, to interpret emotions in their children’s messages and reflect them back in conversational responses; and (6) appropriate requesting, to make requests in a respectful tone instead of ordering (Figure 1).

To improve resilience and reduce depressive symptoms in mothers, strengths-based cognitive behavioral therapy techniques were used [41]. A horticultural therapist identified each mother’s strengths to assist in overcoming stressful situations during the care farming sessions and then helped the mothers to realize those strengths and reinforce them during the care farming activities (Figure 1).

An emotional intelligence model was also incorporated into the care farming program to improve children’s emotional intelligence [42]. Emotional intelligence factors consisted of the following six sequential steps: (1) emotional perception, (2) emotional expression, (3) ability of self-regulation, (4) recognition of other people’s emotions, (5) empathy, and (6) interpersonal relationship skills (Figure 1). These factors were implemented into each session, and the participating children practiced them during the care farming activities.

### 2.3. Implementing the Six-Session Care Farming Program for Family Health

**Figure 2:**
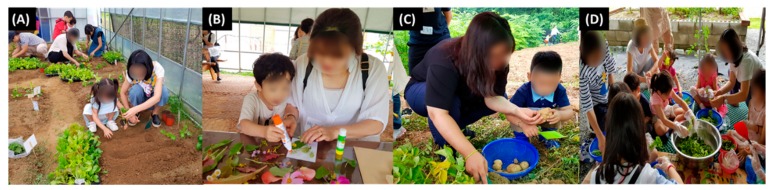
Examples of the care farming activities used in the study (**A**) planting transplants, (**B**) collecting plants, (**C**) harvesting potatoes, and (**D**) cooking the harvests.

The care farming program included six 90 min sessions, once per week from May to July 2018. A care farm located in a semi-urban area of Sejong, South Korea was prepared for this study. The total area of the care farm was 6600 m^2^, and the program used places such as a garden plot (2330 m^2^), greenhouse, outdoor activity area, and kitchen. Each family was provided an individual garden plot (1.5 m × 1.5 m) (Figure 2).

**Figure 3:**
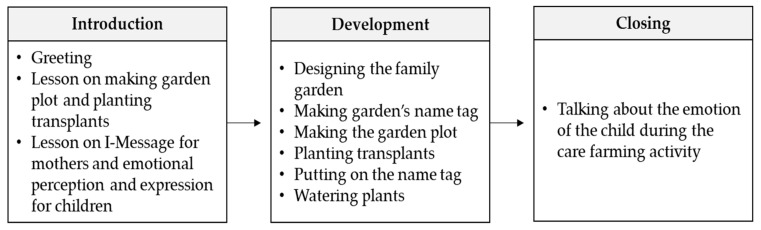
Example of a care farming session for family health.

At the beginning of each session, an instructor explained the topic and lessons of the session and provided a demonstration of the gardening activities. Then, each family was asked to perform the session’s activities. For example, in the second session, mothers and children were asked to plant a family garden together, while accomplishing the goals of mothers using I-messages and children perceiving their own emotions and expressing them. I-messages are a form of assertive interpersonal communication to express feelings, needs, and concerns to others (Figure 1 and Figure 3). For example, if a mother wanted to warn her child and correct his or her misbehavior, she could say, “I am worried about your behavior. I want you to put down the shovel and plant these seedlings with me,” instead of saying, “Drop the shovel and get to work planting these seedlings. You never listen to me!” The children were assigned to perceive their own emotions while planting in the family garden and to talk about their emotions with others at the end of the session (Figure 1 and Figure 3).

To improve resilience and decrease depressive symptoms in mothers, a horticultural therapist identified each mother’s strengths by observing their behaviors and interactions with their children and the other families while working in the family gardens. Then, to encourage the mothers to recognize and reinforce their identified strengths, the therapist complimented behaviors that explicitly displayed those strengths (Figure 1). For example, when the therapist observed that a mother had a good sense of design and created an aesthetically pleasing garden with her child, the therapist said something along the lines of, “You have great gardening skills and pay close attention to details!” The therapist performed this intervention consistently over the program’s six sessions.

In the fifth session of the care farming program, mothers were asked to use the Baby BANK skill, while children were asked to recognize the emotions of others (Figure 1). The Baby BANK skill is a communication technique that helped mothers encourage their children to participate in the program. The children were asked to observe the plants’ appearances (e.g., wilted stems and senescent leaves) and then to infer the plants’ emotions during the activity. Along with this activity, each child was also asked to identify his or her mother’s emotions through her facial expressions and behaviors.

This program was primarily managed by a horticultural therapist who was certified by the Korean Horticultural Therapy and Wellbeing Association. Seventeen volunteers from the Sejong Metropolitan Autonomous City Agriculture and Rural Development Administration assisted in the program.

### 2.4. Assessments

An online survey was developed using Google and conducted to assess improvements in communication skills between mothers and children, depressive symptoms and resilience in mothers, and emotional intelligence in children. The survey included a total of 90 questions, and mothers completed it twice—before and after the six-session care farming program.

To measure participants’ communication skills, a Parents and Children Communication Inventory was used [43,44]. This scale consists of 15 questions scored using a four-point Likert-type scale with the following two subcategories: open communication (10 questions) and negative communication (five questions). Total scores range from 15 to 60, with a higher score indicating better parent-child communication. The Cronbach’s alpha (α) coefficients for the survey were 0.91 and 0.76 for open and negative communication, respectively [45].

To measure depressive symptoms in the mothers, the Korean version of the Beck Depression Inventory (K-BDI) was used [46,47]. This scale consists of 21 questions, scored using a four-point Likert scale. Total scores for the K-BDI range from 0 to 63, with a higher score indicating more severe depressive symptoms. A score ≤ 9 indicates a normal condition, 10–15 indicates mild depression, 16–23 indicates moderate depression, and ≥ 24 indicates severe depression. The Cronbach’s alpha (α) coefficient for the survey was 0.84 [47].

The Korean version of the Connor–Davidson Resilience Scale (K-CD-RISC) [48,49] was used to evaluate resilience in the mothers, which indicates the ability to cope and successfully adapt despite adversity (e.g., risk, stress, or trauma) [50]. The survey included a total of 25 questions scored using a four-point Likert-type scale with the following five subcategories: hardiness, durability, optimism, support, and spirituality. Total scores for the K-CD-RISC range from 0 to 100, with higher scores indicating a higher degree of resilience. The Cronbach’s alpha (α) coefficient of the survey was 0.93 [48].

Emotional intelligence is a form of social intelligence that represents the ability to understand and explain emotions in one’s self and in others [51]. To evaluate the emotional intelligence of the children, the Emotional Intelligence Rating Scale for Preschool Children was used. This rating scale was developed based on an emotional intelligence model by Lee [52]. Using this scale, emotional intelligence was evaluated via a mother’s observation of the daily life of her child. The scale consists of 31 questions scored using a five-point Likert-type scale with the following four subcategories: perceiving emotions (seven questions), managing emotions (eight questions), facilitating thought (nine questions), and understanding emotions (seven questions). Total scores for the scale range from 31 to 155, with a higher score indicating a higher degree of emotional intelligence. The Cronbach’s alpha (α) coefficient of the survey was 0.88 [52].

Additionally, a satisfaction survey [36] was revised and tailored for this study’s care farming program and completed by all mothers and children after the conclusion of the program. The satisfaction survey for mothers included nine questions regarding overall satisfaction with the care farming program, duration, frequency, and activity time per session, preferences regarding care farming activities, benefits of the program, desire to continue participating in the program, and intent to recommend the care farming program to other families. The satisfaction survey for children included four questions regarding interest, difficulty, desire to continue participating in a care farming program, and preferences for performed activities. All participants completed the pre-intervention survey online and the post-intervention survey on paper.

### 2.5. Data Analysis

To compare the results of the measures for the mothers and children before and after the care farming program, the Wilcoxon signed-rank test was conducted using SPSS software (version 24 for Windows; IBM corp., Armonk, NY, USA). Values of *p* < 0.05 were considered statistically significant. Demographic information and satisfaction of mothers and children with the care farming program were analyzed using Excel software (Microsoft Office 2010; Microsoft Corp., Redmond, WA, USA).

## 3. Results

### 3.1. Effects of the Care Farming Program on Communication Skills, Depression, and Resilience in Mothers

The mothers who participated in the six-session care farming program experienced significantly improved mother-child communication skills (pre-test: 44.81 ± 4.40, post-test: 47.44 ± 5.33; *p* = 0.024; Table 2) and resilience (pre-test: 60.13 ± 10.44, post-test: 66.63 ± 12.45; *p* = 0.028; Table 3). Mothers’ depression also significantly decreased from 11.25 ± 6.71 pre-test to 6.25 ± 7.68 post-test and depressive symptoms decreased from mild depression before the program to normal at the end of the program (*p* = 0.003; Table 3).

### 3.2. Effects of the Care Farming Program on Children’s Emotional Intelligence

The children exhibited significantly improved emotional intelligence scores (pre-test: 113.50 ± 12.84, post-test: 119.19 ± 16; *p* = 0.018; Table 4).

### 3.3. Satisfaction of the Participants for the Care Farming Program

The mothers reported being “very satisfied” (68.75%), “satisfied” (25.00%), or “neutral” (6.25%) with the care farming program; no mother reported being “not satisfied.” The mothers were “very satisfied” (43.75%), “satisfied” (12.50%), “neutral” (6.25%), or “not satisfied” (37.50%) with the six-week activity period. In the case of the mothers who reported feeling “not satisfied” (*n* = 6), they responded that a longer period such as 8 weeks, 10 weeks, or 12 weeks would be a more adequate duration. The mothers were also “very satisfied” (62.50%) or “satisfied” (37.50%) with a session frequency of once per week. The mothers were “very satisfied” (43.75%), “satisfied” (37.50%), “neutral” (6.25%), or “not satisfied” (12.50%) with the 120 min activity time per session. In the case of the mothers who reported feeling “not satisfied” (*n* = 2), one respondent commented that a shorter activity time of 90 min would be more adequate and one respondent commented that a longer activity time of 180 min would be more suitable. Additionally, 87.50% of the mothers reported that they felt they benefitted from improved communication with their children through the care farming program. The mothers’ most preferred gardening activities were “harvesting” (16.67%), “cooking the harvested crops” (12.07%), and “planting transplants in the garden” (12.07%). Furthermore, 93.75% of mothers reported they wished to continue participating in the program with their children, and all of the mothers reported they would recommend it to other families.

Moreover, all the children reported they were interested in the care farming program, and 86.66% of children answered that the level of difficulty of the program was suitable. The children’s most preferred gardening activities were “cooking the harvested crops” (16.67%), “collecting the plants” (14.29%), and “watering” (11.09%). Additionally, 93.33% of the children reported they wished to continue participating in the program.

## 4. Discussion

The care farming program for mothers and children presented in this study was found to be effective in improving mother-child communication skills, depressive symptoms and resilience in mothers, and emotional intelligence in children. These results supported our initial hypothesis.

Mothers showed significant increases in mother-child communication skills and resilience and significant decreases in depression in post-tests (Table 2 and Table 3). Mother-child communication skills gained through the care farming program were likely derived from the parenting education lessons that were applied in each session (Figure 1). Mothers practiced parenting skills with their children while carrying out the care farming activities and our findings indicate that joint participation of mothers and children in a care farming program is more effective in improving communication skills than programs that only involve parents [53]. While cultivating or communing with plants on the care farm in this study, the children displayed various behaviors and emotions that provided the mothers with spontaneous chances to practice their parenting skills. As children reacted to their mothers, the mothers also gained immediate feedback on their practices from their children. Moreover, sharing the experience of interacting with nature through a care farming program has been shown to improve mother-child communication skills in previous studies [54,55]. Parenting style affects healthy child development so it is important for parents to learn diverse skills and build a wide knowledge base to perform their parental roles more effectively, especially regarding communicating with and understanding their children [56].

Previous studies have also reported improvements in mother-child communication by participating in care farming activities, similar to our findings [26,27]. In a study by Lee [26], 10 mothers with 7 to 9 year-old children showed significant improvements in mother-child communication in terms of open communication and communicating about problems after a 24 session care farming program, and the effect was retained three months after the program ended. However, this study focused mainly on children and did not fully explore the role of interactions between the mother-child pairs. In our study, we focused on care farming practices that create positive mother-child communication essential for establishing mutual understanding, preventing conflict, and promoting intimacy and closeness in family relationships [57].

The effects on mothers’ psychological health observed in this study resulted from the awareness and reinforcement of their strengths, as promoted by the horticultural therapist in the care farming program (Figure 1). Strength utilization is one of the determinants that can facilitate individual resilience, defined as the ability to adapt in the face of adversity. During care farming activities, the mothers used their identified strengths by making an effort to resolve a stressful situation that could occur in mother-child relationships or relationships with other family members. Resilience is closely related to the management of stress and depression [58]. In this study, recovery of the mothers’ resilience likely helped decrease depression caused by stress.

Moreover, the positive effects observed in this study were likely derived from participants’ interactions with plants in a natural environment [59]. Exposure to nature has been shown to generate psycho-physiological benefits following stress and attention fatigue [60]. Previous studies have reported the positive effects of visual stimulation from or physical involvement with green plants on physiological and psychological relaxation [61], which aligns with the results of the present study. The mothers were thought to gain psychological benefits not only by being exposed to a natural environment but also by actively growing plants on a regular basis. The findings of this study are consistent with those of previous studies [25,62]. A study with the families of 14 children with disabilities who participated in a care farming program for 18 sessions found significant increases in family resilience and adaption compared with control groups [25]. Park and Jung [62] reported that 14 middle-aged women showed significant decreases in depression scores, from 10.57 ± 2.50 to 4.14 ± 1.77, after a 10-session program using plants. Improvements in communication skills and the psychological health of mothers through care farming programs have also been shown to be closely related to improvements in family health [63].

The children showed significant increases in emotional intelligence after participating in the care farming program (Table 4). The positive effects on the children’s emotional intelligence were likely due to the application of emotional intelligence factors such as perception, expression, and recognition of emotions in others in each session of the program (Figure 1). Children’s experiences on care farms or in observing the plant growth process with their mothers provide opportunities for emotional development [36]. In this study, the care farming program provided the children with opportunities to perceive and express various emotions while growing or interacting with plants. By accompanying their mothers, the children could also practice perceiving and sympathizing with their mothers’ emotions during the care farming program. Childhood is a critical period for developing emotional intelligence, which is the ability to recognize, understand, express, and regulate emotions [64,65]. Specifically, to foster emotional intelligence in children, it is important to encourage parents to focus more on their emotional interactions with their children [66]. In the care farming program, the mothers were systematically encouraged to use parenting skills to improve mother-child interactions. This likely worked as an effective intervention that could potentially facilitate the development of children’s emotional intelligence. Previous studies have also reported improvements in emotional intelligence from care farming programs [32,36]. Park et al. [36] reported that a total of 336 children aged five to seven years showed improvements in emotional intelligence after 24 sessions of a care farming intervention.

A healthy family is defined as a family whose members encourage the healthy development of each individual and closely interact with each other [63]. In this respect, the mothers and their children in the care farming program showed characteristics of a healthy family. First, we found improvements in the mothers’ psychological health and children’s emotional intelligence, which assist healthy individual development. Second, we found increased interactions between mothers and children while performing care farming activities. These two findings meet the criteria for the definition of a healthy family; thus, it can be assumed that the care farming program contributed to the improvement of family health.

The present study was designed as a pilot study to elicit and observe effects of a care farming program on family health. The care farming program used in the study is novel in that it differs from the previous care farming programs conducted to date in South Korea by emphasizing the interactions between mother-child as paired participants. However, the methodology employed in this study contains limitations. The results of the care farming program were not conclusive, and the effects of it were exploratory rather than inferential. The nonprobability sampling used in this study means that results cannot be generalized because the families that participated in the program did not accurately represent the statistical population. The one group pre-test-post-test design that was used in the analysis has the least rigorous characteristics. As a result, we were not able to control external factors (e.g., leisure activities of the participant families and participation in other educational events) that reduce the validity of the study, so it is not entirely clear if the positive effects on the family health parameters measured were solely derived from the participation in the care farming program. Thus, the limited interpretation of the study is that the care farming program developed in the study can be improved and expanded upon to perform an appropriate statistical test to validate the effects that were found in the present study. Future studies will be required to address this point.

Future studies should be conducted to evaluate care farming programs with well-balanced settings and control groups in the design. For example, comparing care services with and without farming activities would enable identification of the main factor from which beneficial effects are derived. Additionally, evaluating the individual contributions of each of the various care farming activities to the observed effects would provide informative data. Longer intervention periods are also recommended in future studies, as the six-week period did not allow conclusions to be drawn regarding the results. Finally, future efforts will be required to investigate the effects of care farming programs on individuals from various family types, such as single-parent families, grandparent-headed families, multi-cultural families, and foster/adoptive families.

## 5. Conclusions

The participating mothers and children demonstrated active and positive interactions with each other during the assignment-driven, six-week care farming program. We interpreted these responses as significant improvements in family interactions and psychological family health, which fulfilled the objective of the study. These results suggest that a care farming program with mothers and children participating together could be used as a parent training program for improving family interactions. We identified limitations of this pilot study regarding its design that does not allow to control for possible mixed-effects of external factors and therefore inhibits the inference of causality between the care farming intervention and the observed positive effects. However, given that the existing literature contains few quantitative or qualitative evaluations of care farming for families conducted in South Korea, the present study is significant in that it provides preliminary data on the effects of a care farming program on family health. Care farming programs that promote moderated interactions between family members could potentially be used in South Korea as interventions to address family problems derived from lack of interaction.

## Figures and Tables

**Table 1:** Descriptive information of mothers and children who participated in the care farming program for improving family health (N = 32; 16 mothers, 16 children).

Variable	Care Farming Intervention Group
Age (years)	
Mothers	37.81 ± 3.45 ^1^
Children	3.31 ± 0.95
Number of family members	3.38 ± 0.50
**Mothers**	
Highest education level	
High school diploma	1 (6.25) ^1^
University degree	15 (93.75)
Reported leisure activities (multiple responses possible)	
Exercise	5 (31.25)
Cooking	2 (12.50)
Horticultural activity	2 (12.50)
Religious activity	1 (6.25)
Travel	1 (6.25)
Volunteer	1 (6.25)
Other	3 (18.75)
None	4 (25.00)
**Children**	
Gender	
Boys	7 (43.75)
Girls	9 (56.25)
Reported leisure activities (multiple responses)	
Exercise	4 (25.00)
Playing musical instruments	3 (18.75)
Horticultural activity	3 (18.75)
Cooking	2 (12.50)
Art activity	2 (12.50)
Other	7 (43.75)
None	3 (18.75)

^1^ Data presented as mean ± standard deviation or *n* (%).

**Table 2:** Wilcoxon test comparisons for family interaction before and after the care farming program.

Variable	Pre-test	Post-test	*p* ^1^
	Mean ± SD		
Mother-child communication skills	44.81 ± 4.40	47.44 ± 5.33	0.024 *

^1,^* Significant at *p* < 0.05.

**Table 3:** Wilcoxon test comparisons for mothers’ depression and resilience before and after the care farming program.

Variable	Pre-test	Post-test	*p* ^1^
	Mean ± SD		
Depression	11.25 ± 6.71	6.25 ± 7.68	0.003 **
Resilience	60.13 ± 10.44	66.63 ± 12.45	0.028 *

^1,^** and * Significant at *p* < 0.05 and *p* < 0.01, respectively.

**Table 4:** Wilcoxon test comparisons for emotional intelligence of children before and after the care farming program.

Variable	Pre-test	Post-test	*p* ^1^
	Mean ± SD		
Emotional intelligence	113.50 ± 12.84	119.19 ± 16.30	0.018 *

^1,^* Significant at *p* < 0.05.
